# Computed Tomographic Findings in Dogs with Presumed Metaphyseal Osteopathy

**DOI:** 10.3390/vetsci12090813

**Published:** 2025-08-26

**Authors:** Giulia Dalla Serra, Cliona Skelly, Olga Amorós Carafí

**Affiliations:** 1 Department of Diagnostic Imaging, School of Veterinary Medicine, UCD Veterinary Hospital, University College Dublin, Belfield, Dublin 4, D04 W6F6 Dublin, Ireland; cliona.skelly@ucd.ie; 2 Vet CT Specialists Ltd., Hauser Forum, 21 JJ Thomson Ave, Cambridge CB3 0FA, UK; olga.amoroscarafi@vet-ct.com

**Keywords:** metaphyseal osteopathy, hypertrophic osteodystrophy, computed tomography, dog

## Abstract

Metaphyseal osteopathy (MO) is a rare systemic disease primarily affecting young large-breed dogs. Diagnosis of MO is usually based on a combination of signalment, presenting clinical signs, and imaging findings. While radiographic features of MO are well documented, reports describing Computed Tomography (CT) findings are limited. Clinical records of a veterinary teaching hospital were searched for dogs with a presumed diagnosis of MO that underwent CT imaging. CT and available radiographic studies were retrospectively reviewed. Four dogs met the inclusion criteria. All presented with pyrexia, often associated with other clinical signs. CT lesions were bilateral and symmetric in all cases, characterised by an irregular metaphyseal band of lysis parallel to the physis with associated signs of bone sclerosis. Multiple long bones were affected, and mandibular involvement was suspected in two cases. Comparison with a radiographic study was available in two cases, and follow-up CT imaging was performed in one. Although radiographs remain the primary diagnostic tool for MO, understanding the CT features of the disease is valuable and may contribute to prompt diagnosis, particularly when evaluating young dogs with pyrexia and non-specific clinical signs.

## Simple Summary

Metaphyseal osteopathy (MO) is a rare bone disease that affects young dogs, often presenting with non-specific symptoms such as fever, pain, and lethargy. It is typically diagnosed using radiographs, which show characteristic changes mainly affecting the long bones, and the exclusion of other causes. Following a retrospective review of the hospital records, this case series of four dogs explores the use of Computed Tomography (CT) in assessing bone changes in dogs with presumed MO. Due to CT being a cross-sectional imaging method, improved visualisation of the affected bones helped identify early subtle bony changes and involvement of less commonly affected areas, such as the mandibles. These findings highlight the potential value of CT in supporting an earlier and more accurate diagnosis of metaphyseal osteopathy in dogs. By improving early recognition of this disease, veterinarians may be able to provide faster and more targeted care for affected dogs.

## 1. Introduction

Metaphyseal osteopathy (MO), also known as hypertrophic osteodystrophy (HOD), is a rare systemic orthopaedic disease that primarily affects young (2–8 months old), rapidly growing large- and giant-breed dogs [1,2,3,4,5]. Breeds more commonly affected include Great Dane, Weimaraner, German Shepherd, and Irish Setter [1,2,3,4,5]. However, MO has also been reported in toy and medium-sized breeds and in dogs up to 15 months of age [3,6,7,8]. While no definitive sex predilection has been established, male dogs are overrepresented in several studies [3,7,9]. The aetiology of MO remains unclear, although a multifactorial origin is suspected. Proposed contributing factors include canine distemper virus infection, recent vaccination, hereditary predisposition, autoimmune responses, vitamin C deficiency, and overnutrition [1,3,10,11,12,13]. Clinical signs of MO range from mild and self-limiting to severe, systemic, and potentially life-threatening, with the most common clinical signs including lameness, lethargy, and pyrexia [4,7,9,10,11]. The hallmark clinical feature is typically bilateral, symmetric swelling at the level of the metaphyses of long bones, often associated with localised warmth and pain [2,3,7,9,11]. The distal metaphyses of the radius, ulna, and tibia are most frequently affected, although lesions have also been reported in the mandibles, humeri, ribs, scapulae, vertebral bodies, and metacarpal and metatarsal bones [2,3,9,11,12,14].

Diagnosis of MO is usually based on a combination of signalment, presenting clinical signs, and radiographic findings [4].

Radiographic changes differ based on the stage of the disease but are usually bilateral and symmetric. Reported early radiographic signs include an irregular radiolucent band within the metaphysis and parallel to the physis (also known as “double physis sign”), along with a narrow zone of metaphyseal osteosclerosis and adjacent soft tissue swelling. In more advanced stages, metaphyseal sclerosis with paracortical cuffing separated from the cortex by a radiolucent line may be seen. In the chronic inactive stage, fusion of the metaphyseal collar with the cortex can give the appearance of cortical thickening [1,7,11,15].

While the radiographic features of MO have been well documented, reports on the appearance on Computed Tomography (CT) remain limited, despite the increased availability of this imaging modality. A case report from 2015 was the first describing CT features in a dog with MO [16]. Abnormal findings included bilateral, fairly symmetric metaphyseal lesions in the proximal humerus and distal radius and ulna, characterised by metaphyseal moth-eaten lysis, mild adjacent increased bone attenuation, and surrounding soft tissue swelling [16]. In a more recent study of thirty-nine dogs with MO, four were diagnosed based on CT findings [7]. Those included a hypoattenuating metaphyseal band with or without adjacent bone sclerosis and periosteal reaction, multifocal mottled metaphyseal lysis, and thinning of the central portion of the ulnar growth plate. Affected bones included the humerus, radius, and ulna, with changes noted bilaterally [7].

The aim of this study is to describe CT features in dogs with presumed MO and to contribute to the characterisation of the disease using this advanced imaging technique, addressing the current scarcity of CT-based descriptions in the veterinary literature.

## 2. Materials and Methods

This retrospective, single-centre, descriptive case series was conducted using data from the medical records of the University College Dublin Veterinary Hospital. The hospital’s database was searched to identify dogs with a presumed final diagnosis of MO, based on signalment, history, clinical findings, and imaging features, over the period from November 2009 to December 2024. Owner consent had been obtained for all diagnostic procedures and treatments performed.

Dogs were included in the study if medical records and CT images were available for review. CT examinations had been performed using a 16-slice helical scanner (Siemens SOMATOM Scope). The CT acquisition protocols varied according to the clinical presentation and the anatomical region scanned. One patient underwent repeat CT imaging. Medium- and high-frequency image reconstructions were available for all studies, and post-contrast series were obtained in all but one CT study.

CT images, and when available, radiographic studies, were reviewed by a third-year veterinary radiology resident (G.D.S.) and a board-certified veterinary radiologist (O.A.). Both reviewers were aware of the initial clinical diagnosis of MO at the time of image evaluation. The final decision on the imaging characteristics was reached by consensus.

The diagnosis of MO was based on imaging findings consistent with previously described radiographic and CT features of the disease, in conjunction with the patient’s signalment, clinical presentation, and relevant laboratory test results.

## 3. Results

Four dogs met the inclusion criteria. Detailed information about signalment, clinical and laboratory findings, treatment options, and follow-ups is available in Appendix A Table A1.

### 3.1. Case 1

A 4-month-old, male entire Border Collie was referred for evaluation of a 4-day history of progressively worsening lethargy, reluctance to walk, weakness, and a stiff, stilted gait affecting both pelvic limbs. The dog had been vaccinated two weeks before the onset of the clinical signs. There was no history of travel outside the country. Prior to referral, the dog was pyrexic (rectal temperature 40.1 °C), and haematological analysis revealed leucocytosis with neutrophilia and monocytosis. Due to progression of clinical signs, the dog was referred to our institution.

On physical examination, the dog was quiet but alert and responsive, with mild generalised muscle atrophy and a body condition score of 3 out of 9. Orthopaedic examination revealed pain on carpal flexion and tarsal extension with discomfort upon cervical manipulation. Laboratory tests showed an elevated C-reactive protein (CRP) concentration, consistent with systemic inflammation. SNAP tests for infectious diseases (*Dirofilaria immitis*, *Ehrlichia canis*, *Anaplasma phagocytophilum*, *Angiostrongylus vasorum*, *Neospora caninum*, and *Toxoplasma gondii*) and faecal parasitological screening were negative.

**Figure 1:**
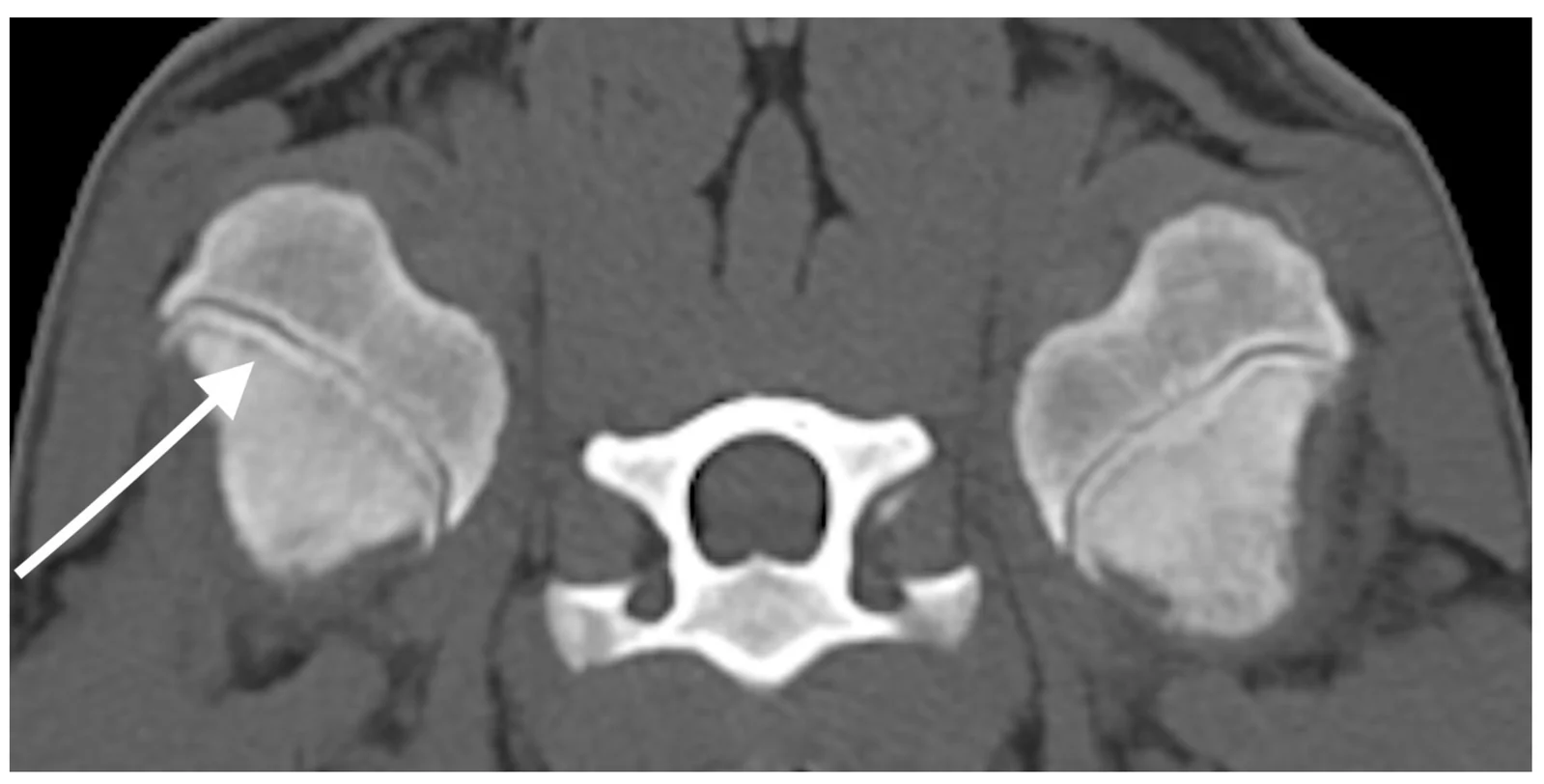
Case 1. Transverse CT image of the proximal humeri with bone algorithm reconstruction at initial presentation. Bilateral symmetric thin hypoattenuating metaphyseal band is present parallel to the physis (white arrow).

**Figure 2:**
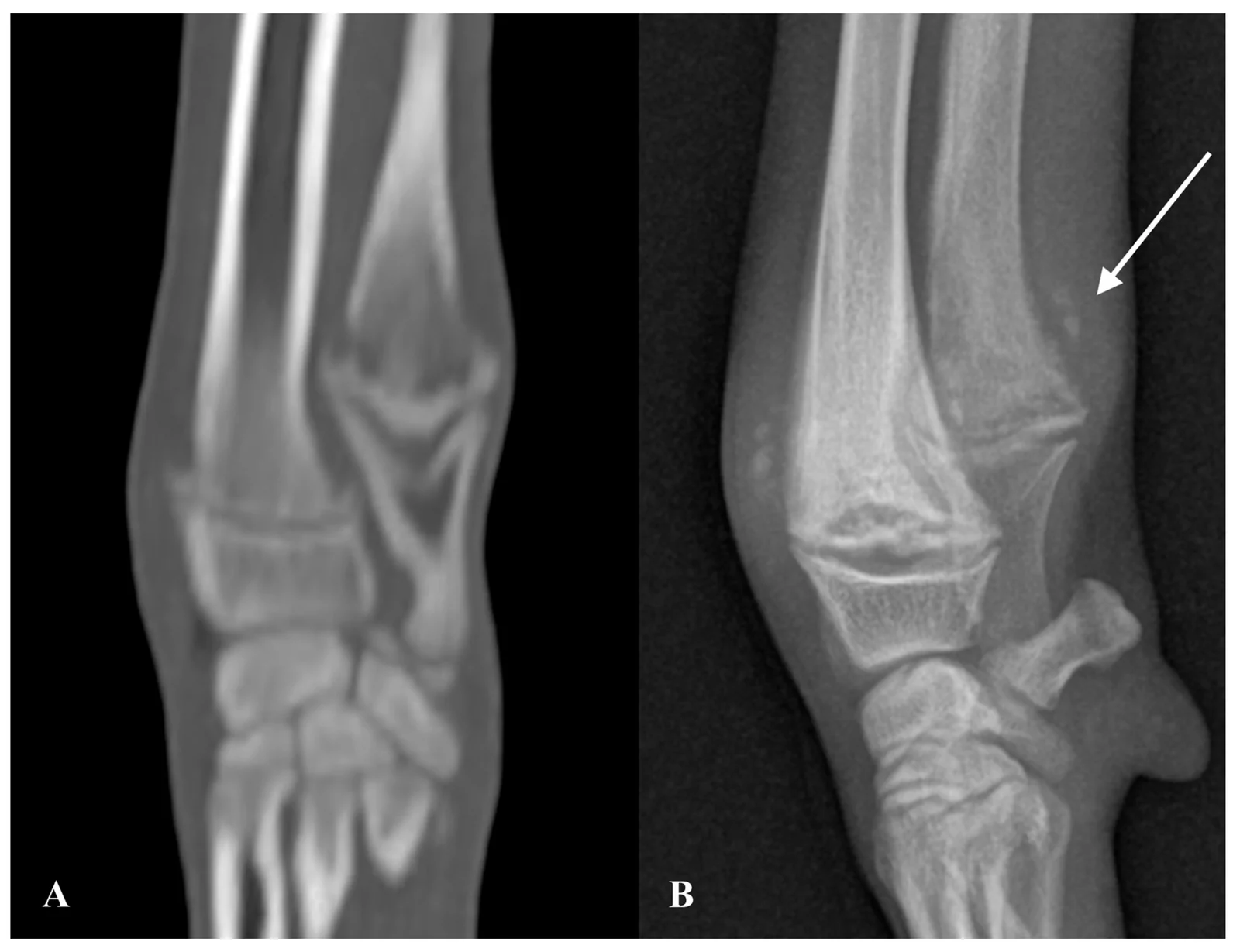
Case 1. Sagittal Multiplanar Reconstruction (MPR) image with bone algorithm of the distal antebrachium on CT at presentation (**A**) and mediolateral radiographic view of the distal antebrachium one month after presentation (**B**). A thin irregular hypoattenuating metaphyseal band is present in the distal radius and ulna, the changes are more severe in the ulna at presentation and mild in the radius (**A**). In the radiograph (**B**), the radiolucent band is associated with adjacent bone sclerosis and mild new bone formation is present cranial to the radius and caudal to the ulna, suspected to represent paracortical cuff (white arrow). Moderate soft tissue swelling centred at the metaphyseal region can also be noted in (**B**).

A full-body CT study was performed before and after intravenous administration of iodinated non-ionic contrast medium. CT findings revealed bilateral, symmetrical metaphyseal changes affecting the proximal humerus, distal radius and ulna, proximal and distal femur, proximal and distal tibia and fibula, and distal metacarpal and metatarsal bones. These changes included an irregular, thin hypoattenuating metaphyseal band of lysis parallel to the physis, associated to a zone of metaphyseal increased attenuation consistent with bone sclerosis (Figure 1 and Figure 2A). The bones were affected to different degrees, some of the changes were very subtle, and the distal ulnae were most severely affected. Very mild new bone formation was incompletely encircling several metaphyses, likely representing normal cutback zones or early paracortical cuffs. No abnormalities were observed within the joints or subchondral bone, and no abnormal contrast enhancement was detected. Mild generalised lymphadenomegaly was present, which was considered a normal age-related finding, or mild reactive hyperplasia. Mild bilateral hypoattenuating foci were present, affecting both mandibular condyles with very mild irregularity of the subchondral bone. The overall imaging findings were suggestive of MO.

Arthrocentesis of the carpal, tarsal, stifle, and elbow joints revealed a predominance of mononuclear cells with a slight increase in non-degenerate neutrophils. However, blood contamination limited interpretation. Cerebrospinal fluid analysis was unremarkable.

Clinical signs improved over the following days with medical treatment, prompting the patient’s discharge.

One month after the initial presentation, the dog re-presented with a relapse of clinical signs, including pain, lameness in all four limbs, and reluctance to walk. Rectal temperature was normal (38.5 °C). Orthopaedic examination identified firm, non-joint-associated swelling at the metaphyses of all long bones. Repeat haematology revealed persistently elevated CRP, although lower than on initial presentation.

Radiographs of the left humerus and bilateral carpi and tarsi revealed an irregular, radiolucent metaphyseal line parallel to the physis within the proximal humerus, and distal radius, ulna, and tibia bilaterally, creating a “double physis sign” (Figure 2B). Mild-to-moderate sclerosis was present adjacent to the radiolucent metaphyseal lines. New bone formation separated from the cortex was present around the affected metaphyses of the radius, ulna, and tibia, consistent with metaphyseal paracortical cuffing, and moderate soft tissue swelling centred on the metaphyses was also noted (Figure 2B).

Although direct comparison with prior imaging was limited due to differences in the imaging modalities, the findings were consistent with progression of the previously suspected MO.

PCR tests for trapped neutrophil syndrome and cyclic neutropenia of the Grey Collie were both evaluated with negative results. Medical treatment was continued, adding steroid therapy at an anti-inflammatory dose to the initial therapy, which was discontinued after two weeks due to significant improvement in the clinical signs and CRP levels.

There was no evidence of an underlying congenital immunodeficiency disorder, and the final presumptive diagnosis was MO.

### 3.2. Case 2

A 6-month-old, male entire Irish Setter was referred to our institution for investigation of a two-week history of pyrexia of unknown origin.

At the time of admission, the dog was bright and alert, with a rectal temperature of 37.9 °C. Pain was elicited on palpation of the lumbosacral spine, with inconsistent and equivocal pain upon joint palpation. The remainder of the physical examination was unremarkable. Laboratory results showed mild lymphocytosis, mildly increased alkaline phosphatase (ALP) activity, and mild hypercholesterolaemia, with urine and blood cultures yielding no bacterial growth.

**Figure 3:**
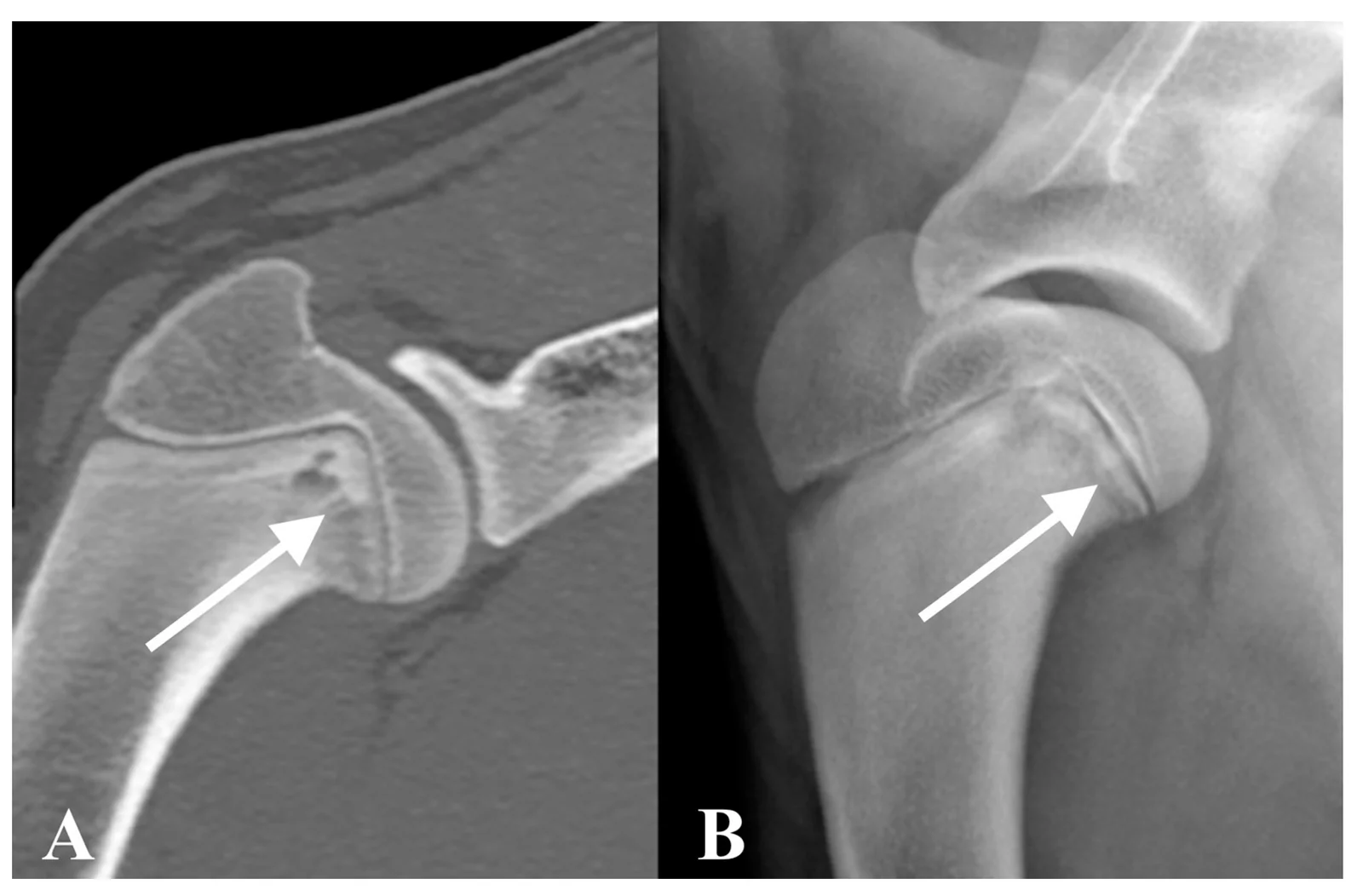
Case 2. Sagittal Multiplanar Reconstruction (MPR) image with bone algorithm of the proximal humerus on CT (**A**) and mediolateral radiographic view of the proximal humerus (**B**). In both images, an ill-defined, irregular lucent band of lysis is present within the proximal humeral metaphysis parallel to the physis (white arrows) with mild associated bone sclerosis.

A CT examination of the neck, thorax, and abdomen was performed before and after intravenous administration of iodinated non-ionic contrast medium. The scan field of view included the proximal forelimbs to the mid-diaphysis of the radius and ulna, as well as the proximal hindlimbs to the distal femurs. CT findings showed bilateral symmetric changes in the proximal humeral metaphysis, consisting of an ill-defined, irregular hypoattenuating band of moth-eaten to permeative lysis parallel to the physis (Figure 3). Adjacent areas of metaphyseal sclerosis were observed, along with mild smooth periosteal new bone formation at the caudoproximal humeral metaphysis and proximal diaphysis bilaterally. The physes appeared normal. Mild generalised superficial lymphadenomegaly was present, which was considered to be a normal age-related finding or mild reactive hyperplasia. The CT findings were suggestive of MO.

Due to the potential need for follow-up imaging, orthogonal radiographs of both distal forelimbs, both stifles, and a mediolateral view of the right shoulder were obtained (Figure 3B). An irregular, radiolucent metaphyseal band parallel to the physis was observed within the proximal right humerus, bilateral distal femur, radius and ulna, and bilateral proximal tibia. Moderate sclerosis of the adjacent metaphyseal bone was present, along with a faint, solid periosteal reaction at the caudal aspect of the proximal right humeral metaphysis. The radiographic findings supported the presumed diagnosis of MO.

The dog was discharged with supportive medical treatment and was lost to follow-up.

### 3.3. Case 3

An 8-month-old, male entire Weimaraner was referred for evaluation of acute onset of spinal pain, pyrexia, bilateral seropurulent nasal discharge, and a confirmed diagnosis of giardiasis.

On presentation, the dog exhibited a low head carriage with reluctance to head elevation. Marked pain was elicited during palpation of the cervical, thoracic, and lumbar spine, as well as during attempts to open the mouth. Rectal temperature was normal, and mild bilateral mandibular lymphadenomegaly was noted. Haematology and biochemistry revealed an elevated CRP concentration, and all other parameters were unremarkable.

A CT examination of the head and neck was performed before and after intravenous administration of iodinated non-ionic contrast medium. The field of view included both shoulders and part of the antebrachia. A post-contrast CT series of the hindlimbs was also acquired following detection of forelimb abnormalities. CT findings included a mild amount of fluid-attenuating material within both nasal cavities, with no associated bony changes, suggestive of mild bilateral secretory rhinitis.

**Figure 4:**
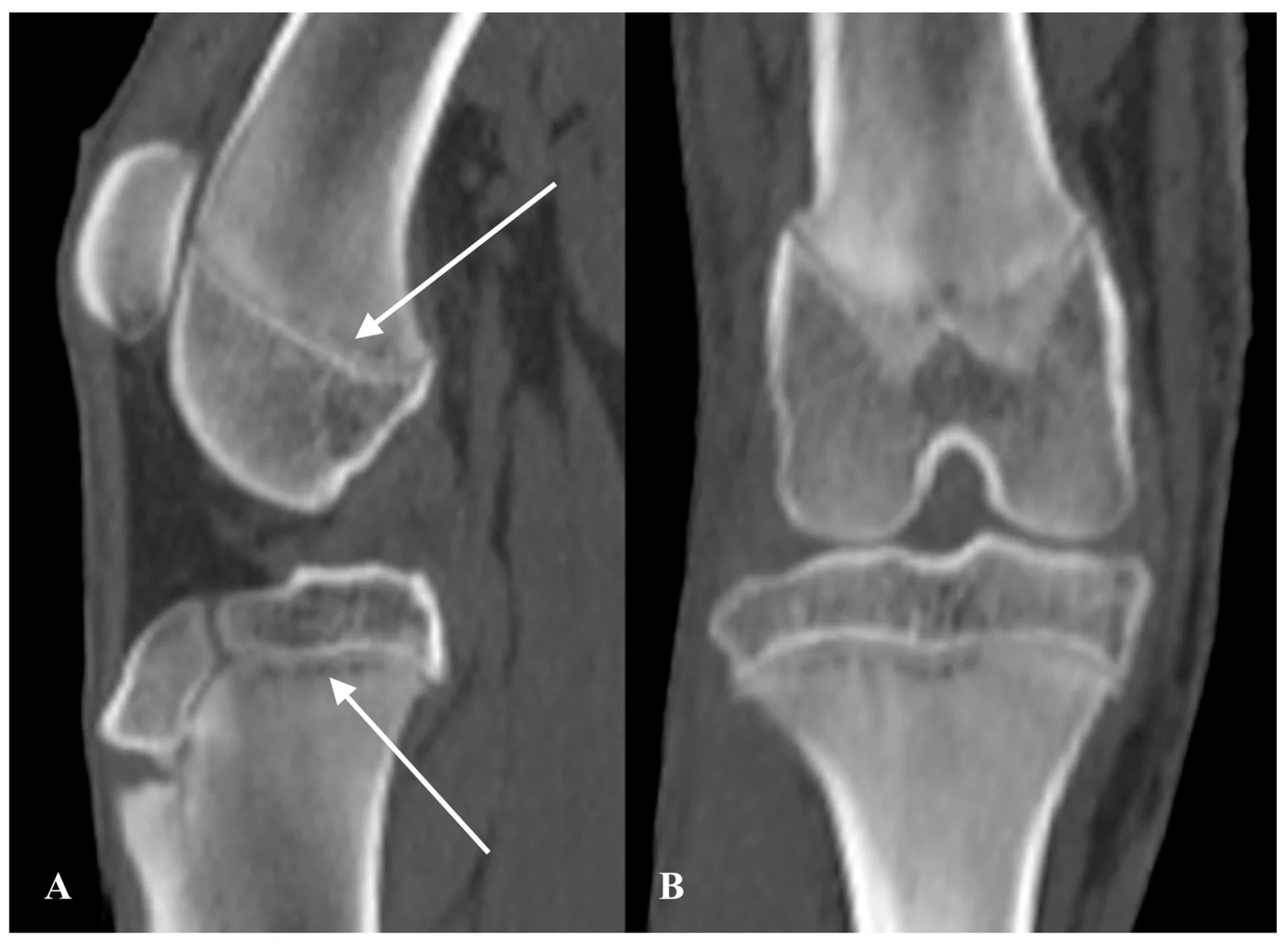
Case 3. Sagittal (**A**) and dorsal (**B**) MPR CT images of the right stifle. Both the distal femoral and the proximal tibial metaphyses show an irregular thin hypoattenuating band (white arrows) with adjacent increased bone attenuation, consistent with osteosclerosis.

The included portions of appendicular skeleton showed bilateral, symmetric, irregular hypoattenuating metaphyseal bands of lysis bordered by sclerotic bone, located parallel to the physes of the proximal humerus, distal radius and ulna, distal femur, and proximal and distal tibia (Figure 4). These findings were suggestive of MO. Mild generalised superficial lymphadenomegaly was noted and was suggestive of a normal age-related finding or mild reactive hyperplasia. The remaining structures were normal.

Cerebrospinal fluid analysis revealed a marked, mixed pleocytosis, predominantly neutrophilic and mononuclear, non-septic in nature. Considering these findings, a concurrent process such as steroid-responsive meningitis–arteritis (SRMA) or meningomyelitis of unknown origin was suspected. The patient was discharged with medical treatment, and a follow-up visit after three weeks revealed a complete resolution of the clinical signs with normal CRP levels.

### 3.4. Case 4

A 3-month-old, female entire Labradoodle was referred for investigation of a 4-day history of lethargy, jaw pain, pyrexia, weakness, and reluctance to walk. The dog had been vaccinated three weeks before the onset of clinical signs.

On physical examination, the dog had a body condition score of 3 out of 9 and mild generalised muscle atrophy. Rectal temperature was 39.3 °C. A stiff gait and generalised weakness affecting all four limbs were observed. Pain was elicited upon manipulation of all limbs and during mouth opening, and the remainder of the physical examination was unremarkable. Laboratory results revealed a moderate non-regenerative anaemia, marked monocytosis, mildly increased ALP, mild-to-moderate hypercholesterolemia, mild hypouremia, and elevated CRP levels.

**Figure 5:**
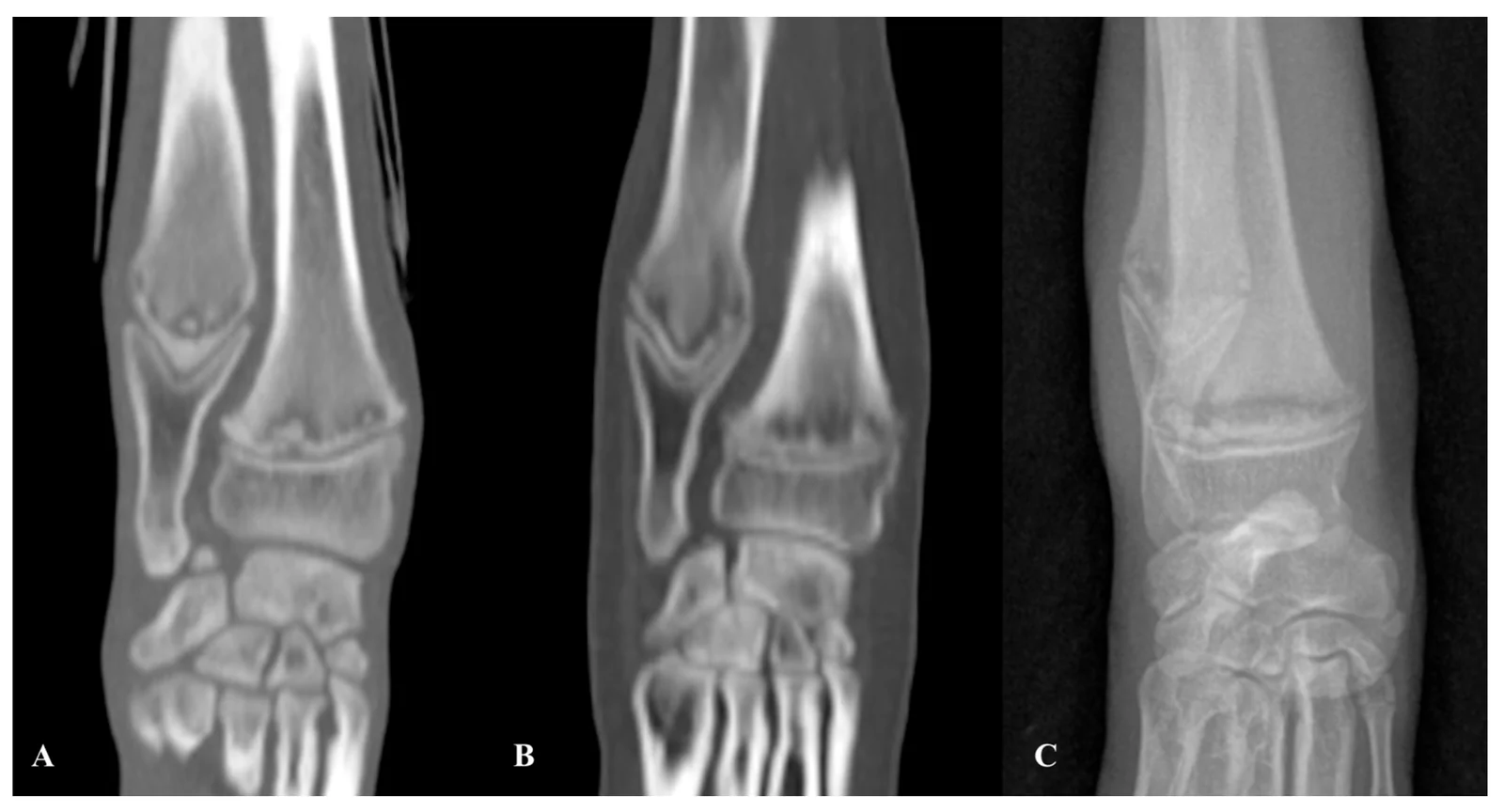
Case 4. Dorsal MPR images reconstructed with bone algorithm of the distal radius and ulna on CT at presentation (**A**) and after 5 weeks (**B**). Craniocaudal radiographic image of the distal radius and ulna at 5 weeks after presentation (**C**). In (**A**), an ill-defined, irregularly marginated lucent metaphyseal band parallel to the physis is present in the distal radius and ulna, with concurrent multiple rounded hypoattenuating lesions with sclerotic centres. In (**B**,**C**), the rounded lesions are less defined and coalescing together, forming a lytic band.

**Figure 6:**
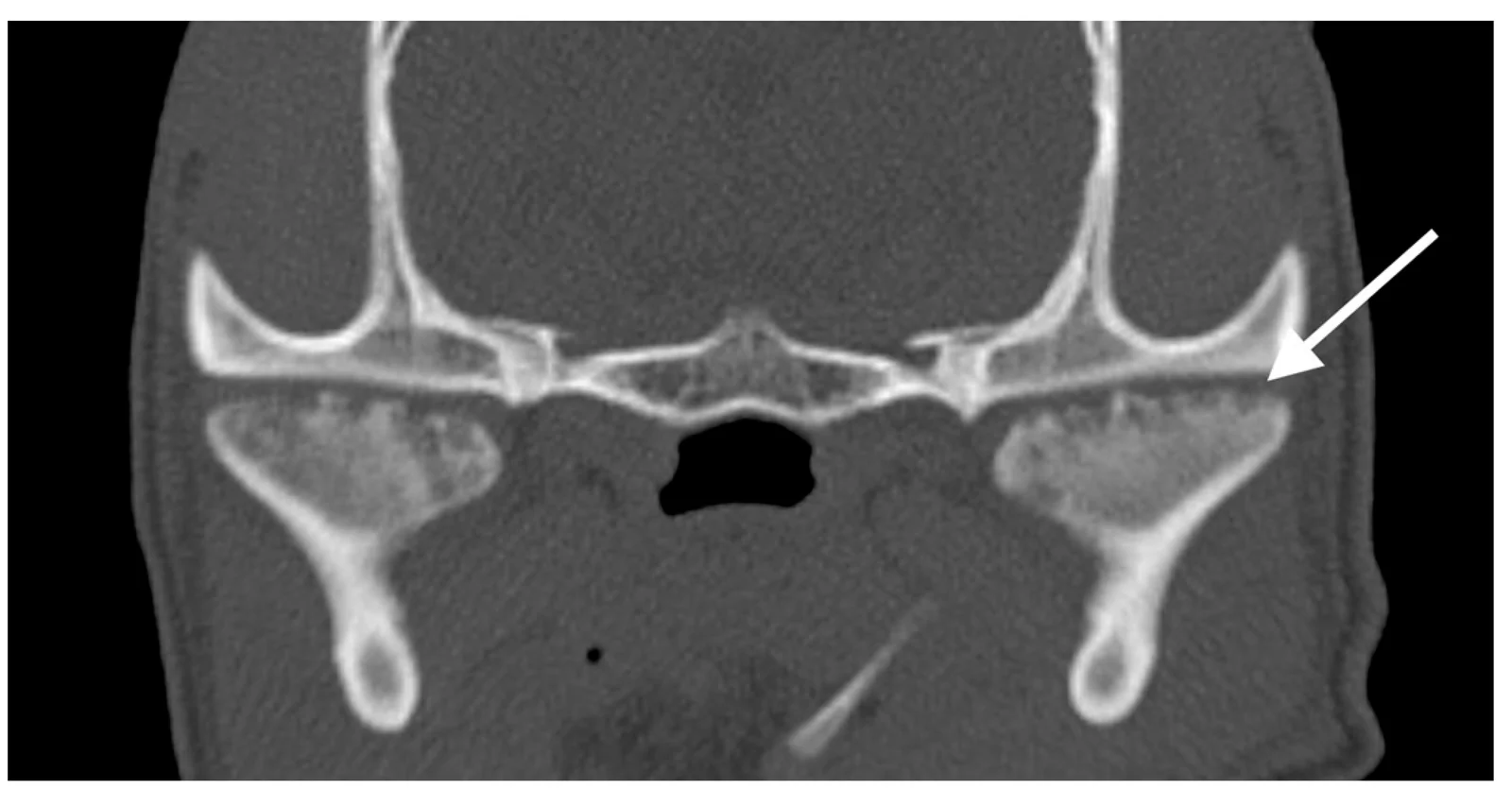
Case 4. Transverse CT image of the temporomandibular joints with bone algorithm reconstruction. Several hypoattenuating rounded lesions, some of which have a focal sclerotic centre, are present in the mandibular condylar processes bilaterally, extending to the articular surfaces (white arrow).

CT examination of the head and forelimbs was performed. Bilateral symmetrical metaphyseal changes were present in the proximal humerus, distal and proximal radius, distal ulna, and distal metacarpal bones. These changes were characterised by ill-defined, irregular hypoattenuating metaphyseal lytic bands parallel to the physis, with concurrent multiple rounded to semicircular hypoattenuating lesions with sclerotic centres (Figure 5A). An ill-defined sclerotic band parallel to the physis was also observed. Mild smooth paracortical new bone formation along the caudoproximal humeral metaphysis was present bilaterally. Mild dilation of the synovial joint with fluid was present at the shoulder and elbow bilaterally. In addition, several hypoattenuating rounded lytic lesions, some of which had a focal sclerotic centre, were present in the mandibular condylar processes bilaterally, extending to the articular surfaces (Figure 6). Moderate generalised superficial lymphadenomegaly was noted and was suggestive of a normal age-related finding or mild reactive hyperplasia. The CT findings in the appendicular skeleton were suggestive of MO with suspect concurrent involvement of the mandibles.

The dog was discharged with medical treatment but re-presented five weeks later due to suspected relapse after stopping treatment. The dog was non-ambulatory, with severe pain in all limbs and upon opening the mouth, along with marked swelling of multiple metaphyseal regions. Rectal temperature was 40.8 °C. Repeated laboratory tests did not reveal significant changes compared to previous results.

A repeat full-body CT scan before and after intravenous administration of iodinated non-ionic contrast medium was conducted. Similar ill-defined, irregular hypoattenuating lytic bands parallel to the physes were present, affecting the same metaphyses within the forelimbs. The previously described hypoattenuating metaphyseal rounded lesions were coalescing together in a more extensive and less defined lytic band. A mild smooth-to-spiculated periosteal reaction was present along the craniodistal aspect of the radial metaphysis. Similar metaphyseal changes were present in the hindlimbs, affecting the proximal and distal femur, tibia and fibula, and distal metatarsal bones bilaterally. Effusion within several joints was again noted, and diffuse subcutaneous fluid attenuation was present along the forelimbs and hindlimbs, suggestive of subcutaneous oedema or cellulitis. The mandibular condylar lytic lesions were reduced in size and less distinct. Moderate enlargement of several superficial and deep lymph nodes was present, along with mild pleural and peritoneal effusions, suggestive of inflammatory or age-related findings. The CT images were suggestive of mild progression of the suspected MO (Figure 5B).

Radiographs were performed to allow for future monitoring. Mediolateral views of the shoulders and orthogonal views of the carpi, stifles, and tarsi revealed bilateral metaphyseal lucent bands parallel to the physis in the proximal humerus, distal radius and ulna, distal femur, and proximal and distal tibia and fibula (Figure 5C). Perilesional sclerosis and moderate soft tissue swelling were also noted. The patient was discharged with medical treatment, with significant improvement in the clinical signs at one-month follow-up.

## 4. Discussion

This study describes the imaging findings of four dogs with presumed diagnosis of MO based on CT images. In all cases, bilateral, irregular, hypoattenuating metaphyseal bands of lysis were observed, running parallel to the physis. These lesions were symmetric, often bordered by areas of metaphyseal sclerosis, and commonly associated with mild periosteal new bone formation. Despite the consistent nature of these imaging features, the severity of the lesions varied both within individual patients and across the different cases. Subtle changes, such as those shown in Figure 1, may represent early manifestations of MO and are unlikely to be detectable on plain radiographs.

Three of the four dogs in this case series belonged to breeds previously reported to be affected by MO [1,4,5,7]. The fourth dog was a Labradoodle, a cross between a Labrador Retriever and a Poodle. While there are no prior reports specifically describing MO in Labradoodles, both Labrador Retrievers and cross-breed dogs have been documented in the literature as affected by MO [5,9,11,14]. Three of the four dogs included in this study were male, which is consistent with previous publications, where male dogs were overrepresented [1,7,9]. The age at presentation ranged from 3 to 8 months, also aligning with the reported age range for MO [1,9].

In all cases, the diagnosis of MO was based on signalment, history, clinical signs, and characteristic imaging findings. Despite reported possible overlap between haematogenous osteomyelitis and MO in their clinical and radiographic presentation [17], haematogenous osteomyelitis was considered a much less likely differential diagnosis due to the presence of the characteristic transverse metaphyseal lytic band and the bilaterally symmetrical presentation of the lesions in the cohort of patients in this study.

Case 1, a Border Collie, was tested and found PCR-negative for trapped neutrophil syndrome (TNS), a hereditary disease known to affect this breed. TNS presents with clinical signs such as pyrexia, abnormal gait, and gastrointestinal symptoms. In some TNS-affected dogs, imaging findings consistent with MO have been reported, particularly involving the distal radius and ulna [18,19]. 

Canine leucocyte adhesion deficiency (CLAD) is another inherited immunodeficiency that primarily affects Irish Setters and their crosses. CLAD is characterised by recurrent infections, neutrophilia, and skeletal abnormalities, including changes consistent with MO, craniomandibular osteopathy, and osteomyelitis [20,21]. Given the breed of case 2 (Irish Setter), genetic testing for CLAD was recommended but the dog was unfortunately lost to follow-up.

Few other conditions can explain metaphyseal bone changes in young dogs. In Newfoundland dogs, incidental metaphyseal remodelling has been described, although these changes were incidental and not associated with clinical signs [22]. Given the breed differences, the severity of clinical signs, and the imaging findings of the patients in this study, this condition was excluded as a possible diagnosis.

Cases 3 and 4 presented with pain on opening the mouth as part of the clinical signs, and case 4 exhibited bilateral lytic lesions in the mandibular condyles. Similar but much less severe changes were also present in case 1. Mandibular involvement, including discomfort when opening the mouth, has been previously reported in association with MO [4,11,12,23]. Lesions involving the mandibular condyles, similar to those observed in case 4, have been described in a dog with MO evaluated by CT [7]. In another case report, a dog with MO and marked bilateral pain on palpation of the caudal mandibles showed similar histopathological changes in both long bones and the mandibular condyles, characterised by fibrinosuppurative metaphyseal osteomyelitis [23]. Considering the similarity of the mandibular lesions to the lesions affecting the appendicular skeleton and the progression detected at the follow-up CT, a mandibular involvement of MO was suspected in case 4. The reported changes in case 1 were more subtle, and possible differential diagnoses included MO, normal anatomical age-related findings, or small subchondral bone cysts. Detecting bilateral mandibular changes on CT in young dogs evaluated for pyrexia of unknown origin should raise suspicion for MO. Subsequent assessment of the metaphyses of the long bones may aid in the early recognition of the disease, even if orthopaedic signs are not yet present.

This study has several limitations. Due to its retrospective nature, the CT acquisition protocol was not standardised, and not all limbs were imaged in each patient. Furthermore, not all dogs underwent radiographic evaluation, which limited direct comparison between CT and radiography. Follow-up CT imaging was available for only one case, limiting the assessment of the temporal evolution of the lesions with this modality. The small sample size is another limitation, although it reflects the rarity of this condition.

Although histopathological confirmation was not obtained, the diagnosis of MO in these cases was supported by a combination of signalment, clinical signs, and characteristic imaging features, consistent with diagnostic approaches used in the previous literature.

## 5. Conclusions

MO is a complex and likely multifactorial disease that can present with variable clinical signs and may coexist with other systemic or genetic conditions [1,3,10,11,12,13]. In affected patients, diagnostic imaging plays a crucial role in establishing the diagnosis. Recognition of characteristic CT features of MO is important, especially when interpreting CT scans of young dogs performed for other suspected clinical indications. While radiography remains the first-line imaging modality for diagnosing MO, CT can be a valuable adjunct—especially in cases presenting with non-specific clinical signs—by likely enabling early diagnosis. As a cross-sectional modality, CT allows multiplanar reconstructions and eliminates superimposition of structures, facilitating the detection of mild bony changes that may not be visible on plain radiographs. Its rapid acquisition time also permits the simultaneous evaluation of multiple body regions, assisting in the identification of concurrent conditions and the exclusion of differential diagnoses. In patients undergoing CT for pyrexia of unknown origin or neurological signs, careful evaluation of included portions of the limbs—such as proximal humeral metaphyses in cervical spine studies—may reveal characteristic changes suggestive of MO.

This study contributes to the limited existing literature on CT features of dogs with MO, expanding the current understanding of the imaging spectrum of this rare disease.

## Data Availability

The original contributions presented in this study are included in the article. Further inquiries can be directed to the corresponding author.

## Abbreviations

The following abbreviations are used in this manuscript:

ALP	Alkaline phosphatase
CRP	C-reactive protein
CT	Computed Tomography
MO	Metaphyseal osteopathy
MPR	Multiplanar reconstruction
PCR	Polymerase chain reaction
TNS	Trapped neutrophil syndrome

## Appendix A

**Table A1:** Detailed information on signalment, clinical presentation, laboratory findings, treatments, and follow-up outcomes.

Case	Symptoms	Recent Vaccination	Physical Exam	Laboratory Findings	Additional Tests	Treatment	Follow-Up
1	Acute and progressive lethargy, reluctance to walk, weakness, and stiff, stilted gait affecting the pelvic limbs.	2 weeks prior the onset of clinical signs.	Pyrexia, quiet but alert and responsive, mild generalised muscle atrophy, BCS 3/9. Pain on carpal flexion and tarsal extension, discomfort upon cervical manipulation.	Leucocytosis with neutrophilia and monocytosis. Elevated CRP.	Negative SNAP tests: *Dirofilaria immitis*, *Ehrlichia canis*, *Anaplasma phagocytophilum*, *Angiostrongylus vasorum*, *Neospora caninum*, and *Toxoplasma gondii*. Negative faecal parasitological screening. Arthrocentesis carpus, tarsus, stifle, and elbow: predominance of mononuclear cells with a slight increase in non-degenerate neutrophils (suspected blood contamination). CSF normal. Negative PCR testing for trapped neutrophil syndrome and cyclic neutropenia of the Grey Collie.	Initial treatment with paracetamol and buprenorphine followed by paracetamol and gabapentin. Corticosteroid therapy added after relapse.	Relapse 1 month later. Clinical signs: lameness in all four limbs, reluctant to ambulate. Physical examination: firm, non-joint-associated swelling at the metaphyses of all long bones. Persistently elevated CRP.
2	Pyrexia.	Unknown.	Pain at palpation of lumbosacral spine, inconsistent pain upon joint palpation.	Mild lymphocytosis, mildly increased ALP, mild hypercholesterolaemia. No bacterial growth in urine and blood cultures.		Meloxicam and paracetamol.	Lost to follow-up.
3	Acute onset spinal pain, pyrexia, bilateral seropurulent nasal discharge. Recent confirmed diagnosis of giardiasis.	Unknown.	Low head carriage with reluctance to head elevation. Marked diffuse pain at spinal palpation. Pain at opening the mouth. Mild bilateral mandibular lymphadenomegaly.	Elevated CRP.	CSF: marked, mixed pleocytosis, predominantly neutrophilic and mononuclear, non-septic.	Prednisolone, paracetamol, and gabapentin. Fenbendazole to treat the concurrent giardiasis.	Follow-up visit after three weeks: complete resolution of the clinical signs with normal CRP levels.
4	Lethargy, jaw pain, pyrexia, weakness, and reluctance to walk.	3 weeks prior the onset of clinical signs.	BCS 3/9, mild generalised muscle atrophy. Rectal temperature = 39.3 °C. Stiff gait and generalised weakness affecting all four limbs. Pain upon manipulation of all limbs and during mouth opening.	Moderate non-regenerative anaemia, marked monocytosis, mildly increased ALP, mild-to-moderate hypercholesterolemia, mild hypouremia, and elevated CRP.		Paracetamol and meloxicam. Corticosteroids were added after the relapse.	Relapse after 5 weeks: BCS 2/9, with persistent mild muscle atrophy. Rectal temperature = 40.8 °C. Non-ambulatory due to severe pain in all limbs. Marked swelling over the affected metaphyseal regions. Severe pain on opening the mouth.

Abbreviations: CRP, C-reactive protein; CSF, cerebrospinal fluid; BCS, body condition score.
